# Five key attributes can increase marine protected areas performance for small-scale fisheries management

**DOI:** 10.1038/srep38135

**Published:** 2016-12-01

**Authors:** Antonio Di Franco, Pierre Thiriet, Giuseppe Di Carlo, Charalampos Dimitriadis, Patrice Francour, Nicolas L. Gutiérrez, Alain Jeudy de Grissac, Drosos Koutsoubas, Marco Milazzo, María del Mar Otero, Catherine Piante, Jeremiah Plass-Johnson, Susana Sainz-Trapaga, Luca Santarossa, Sergi Tudela, Paolo Guidetti

**Affiliations:** 1 Université Côte d’Azur, CNRS, FRE 3729 ECOMERS, Parc Valrose 28, Avenue Valrose, 06108 Nice, France; 2 Consorzio Interuniversitario per le Scienze del Mare, CoNISMa, Piazzale Flaminio 9, 00196 Rome, Italy; 3 WWF-MedPO, Via Po 25/c, 00198 Roma, Italy; 4 Department of Marine Sciences, School of Environment, University of the Aegean, Mytilini, Greece; 5 National Marine Park of Zakynthos, Zakynthos, Greece; 6 Food and Agriculture Organization of the United Nations, Viale Delle Terme di Caracalla, Rome, Italy; 7 IUCN Center for Mediterranean Cooperation, C/Marie Curie 22, 29590 Campanillas, Málaga, Spain; 8 DiSTeM—Department of Earth and Marine Sciences, University of Palermo, Via Archirafi 28, 90123 Palermo, Italy; 9 WWF-France 1 Carrefour de Longchamp, 75016 Paris, France; 10 WWF Mediterranean Programme, Barcelona 08002, Spain; 11 Federparchi – Europarc Italy, Rome Italy

## Abstract

Marine protected areas (MPAs) have largely proven to be effective tools for conserving marine ecosystem, while socio-economic benefits generated by MPAs to fisheries are still under debate. Many MPAs embed a no-take zone, aiming to preserve natural populations and ecosystems, within a buffer zone where potentially sustainable activities are allowed. Small-scale fisheries (SSF) within buffer zones can be highly beneficial by promoting local socio-economies. However, guidelines to successfully manage SSFs within MPAs, ensuring both conservation and fisheries goals, and reaching a win-win scenario, are largely unavailable. From the peer-reviewed literature, grey-literature and interviews, we assembled a unique database of ecological, social and economic attributes of SSF in 25 Mediterranean MPAs. Using random forest with Boruta algorithm we identified a set of attributes determining successful SSFs management within MPAs. We show that fish stocks are healthier, fishermen incomes are higher and the social acceptance of management practices is fostered if five attributes are present (i.e. high MPA enforcement, presence of a management plan, fishermen engagement in MPA management, fishermen representative in the MPA board, and promotion of sustainable fishing). These findings are pivotal to Mediterranean coastal communities so they can achieve conservation goals while allowing for profitable exploitation of fisheries resources.

Across the globe marine fisheries employ 200 million people and provide a primary source of food for one billion people^1^. The importance of fish as food and for jobs has resulted in long-term overfishing of global fish stocks. Currently, 77% of global fish stocks are overfished and this is predicted to increase to 88% by 2050^2^,^3^. The primary consequences of overexploitation are (i) severe environmental impact (from single stocks to ecosystems)^4^ and (ii) substantial public subsidies for the continuation of the fisheries because of their serious socio-economical underperformance^5^. Overfishing leads to a classic “lose-lose” system where ecosystems, economies and the social well-being of people are negatively affected^1^. However, the implementation of fisheries management strategies has resulted in some fish stocks showing encouraging signs of rebuilding^6^. Further benefits are expected if additional management reforms to global fisheries are applied. Some ‘best case’ scenarios suggest that 98% of fish stocks can be rebuilt by 2050 if adequate reform is enacted quickly^2^.

Despite the variety of fisheries management strategies, traditional single-species management strategies are often employed for large-scale fisheries^7^. Single-species management strategies are inadequate for small-scale fisheries (SSF) where the gear and the target species can be temporally and spatially changing. Moreover, due to the complexity of these fisheries, data are generally missing^8^. The limited available data on SSF suggest that 44–60% are overexploited with predictions of the future being unfavorable^6^. The overexploitation of SSF is of critical concern because they represent about 20% of global catches^9^ and play a direct role in securing food and livelihoods for coastal communities^10^. SSFs globally are responsible for half of the catches intended for human consumption and, compared to industrial fisheries, offer more equitable economic and social benefits (e.g., higher employment) to stakeholder communities^11^. However, only recently governments and international organisations (e.g., FAO) have recognised the important contribution of SSF to poverty alleviation, food security and the sustainable blue-economy^10^,^12^.

The failure of current practices to adequately manage fisheries has led to the “Ecosystem Approach to Fisheries (EAF)”^13^, aiming to balance ecosystem health with socio-economic needs. EAF has been identified as the guiding principle to reach sustainability in SSF^10^, while marine protected areas (MPAs) are considered one of EAF’s important elements^14^. In the last decade, the rapid pace of MPA creation has led to 12,000 MPAs and 12 million km^2^ of protected ocean, globally^15^. MPAs are generally multiple-use areas aiming to protect natural populations, ecosystems and the goods and services they provide to society^16^. In concert with protection, MPAs can potentially enhance local fisheries^17^, in particular SSFs, and promote local socio-economies through sustainable development. More than 18% of global MPAs contain both no-take zones (NTZ), where all extractive activities are forbidden and where the focus is on conservation, and buffer zones (BZ), where SSF and other potentially sustainable activities are allowed^18^. The proportion of MPAs containing both NTZ and BZ can be higher in locations where high human densities result in intense use of the sea. A primary example of this is the Mediterranean Sea where about 92% of MPAs contain both NTZ and BZ^19^.

MPAs have been proven an effective tool to achieve conservation goals by allowing the recovery of marine populations and ecosystems^20^,^21^,^22^. However, the role of MPAs in providing fisheries benefits is still debated because the results are often context-dependent^23^,^24^. Although MPAs are not always a ‘one-size-fits-all’ solution^24^,^25^, the benefits of MPAs to SSFs can be substantial since they protect fish stocks in the NTZ and can promote density dependent spill-over processes^19^,^26^ enhancing fisheries catches in the BZ and outside the MPAs^24^,^27^,^28^. MPAs could also play a crucial role in SSF management. Being a spatially explicit conservation/management tool, MPAs make it easier for decision-making systems to cope with the patchy and heterogeneous nature of SSF fisher communities by allowing for the implementation of fisheries regulations that address needs at a localised scale. Furthermore, MPAs provide opportunities to test fishing management practices aimed at sustainability (e.g., fishermen engagement in management^29^).

The use of MPAs can potentially create a “win-win” situation where the challenges of conservation and SSF management can be resolved in parallel^5^,^28^,^30^. However if benefits are over-stated and expectation are not reached, negative stakeholder attitude can reduce compliance creating a negative cycle that further impairs the performance of MPAs^31^. Therefore it is crucial to identify and highlight the characteristics (e.g. environmental, economic and social attributes) that underline success of SSFs management within MPAs, and leading to a win-win scenario.

The management of SSFs within MPAs has received little attention in the scientific literature. Subsequently, little is known of the characteristics that lead to a successful SSF-MPA partnership. Studies on the performance of MPAs generally focus on either conservation (reserve effects^20^,^21^,^22^) or fishery management (enhanced fishery yields^24^,^27^,^32^) independently, neglecting to consider MPAs, SSF and fishing resources as complex socio-ecological system (SES) that integrates both natural and human components^33^,^34^. There are, however, a few exceptions within a tropical context where SSF and fishing resources are analysed in a SES-framework^35^,^36^.

To address this gap in the literature, we identify key characteristics associated with successful SSFs management in Mediterranean MPAs by assembling a unique database of peer-reviewed literature, grey-literature and a set of interviews across 25 MPAs. These MPAs span five countries, cover approximately 3,160 km^2^ and harbour more than 1,000 SSF vessels (Fig. 1, Supplementary Table S1). Small-scale fisheries are fundamental to the economies and societies of the Mediterranean Sea where they employ more than 137,000 fishermen^37^. Although there exists a relatively high number of MPAs in the Mediterranean Sea^19^ (170), 85% of fish stocks are overfished^38^ suggesting that traditional fisheries management has been ineffective^38^,^39^.

The MPAs included in this study are distributed along the central-north region of the Mediterranean basin and they encompass a variety of environmental (i.e. they are located in 4 different ecoregions), management (i.e. MPA size, fishing restrictions, enforcement level, level of fishermen engagement into management) and socio-economic (i.e. countries, presence of a leader among fishermen, fishermen grouped in associations) conditions. Therefore, our findings are relevant throughout the central-north Mediterranean Sea where 96% of all the Mediterranean MPAs are located^19^.

We followed Ostrom’s framework^33^ to identify characteristics of SES and define the success^34^ of SSF management in MPAs. Specifically, for each MPA we extracted a set of predictive variables (i.e. attributes) that describe multiple aspects of SSFs management (see Supplementary Table S2 for a full list and description). Additionally, we identified responses, or outcomes (Table S2), that cover ecological, social and economic aspects of SSF management, a common practice when assessing variables that affect successful management of common pool resources^35^,^40^,^41^. The outcomes included: a) ecological effectiveness (i.e., fish assemblages with higher density/biomass within an MPA compared to unprotected areas), b) economic benefits for fishermen (i.e., higher incomes when fishing within the MPA buffer zone compared to outside) and c) the commitment of fishermen to the environment (i.e., fishermen comply with MPAs rules and participate to research and environmental programs). The three outcomes, defined on a binary scale denoting presence or absence (1 or 0 respectively), were added to build a compound score^34^ termed ‘overall management success (OMS)’. OMS ranges from 0 to 3, or from total failure to high success, and captures the well-being of SSF coastal system, including both human and non-human elements. Finally, we assessed the strength and significance of the correlative relationships between the OMS, the three outcomes, and the set of attributes in order to highlight the circumstances that determine successful win-win management.

## Results and Discussion

Sixty-four percent of MPAs showed ecological effectiveness, 68% showed economic benefits for fishermen and 60% showed commitment of fishermen to the environment (hereinafter “fishermen environmental commitment”). Overall, 40% of MPAs were highly successful (OMS = 3). Given the substantial proportion of unsuccessful MPAs (36% with OMS = 0 or 1), we highlight that establishing MPAs *per se* does not suffice to solve ecological, economic and social challenges related to SSF management. When the goals of an MPA are not met, and the potential benefits are not obtained, stakeholder’s support towards MPAs can be negatively influenced, potentially leading to a negative attitude and reduction in compliance^31^.

This study identified key attributes of MPA and SSFs that can achieve conservation goals while at the same time maintaining profitability in SSFs. These attributes can also drive Blue Growth (i.e. sustainable ocean-based economy) and contribute positively to targets required at the international level^10^,^12^. The identification of key attributes is particularly crucial considering the number of underperforming MPAs^22^.

**Figure 2:**
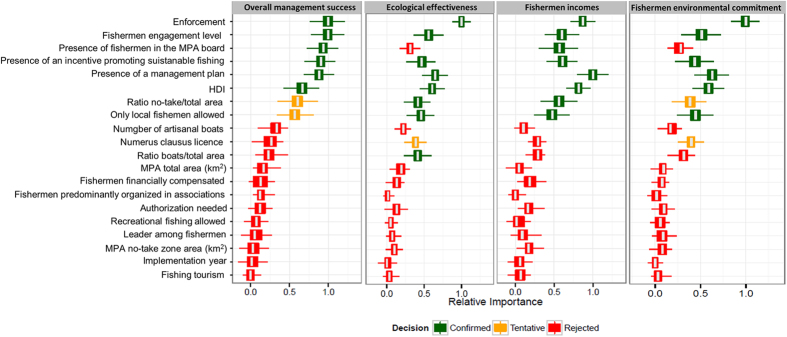
Key features for small scale fisheries overall management success. Boxplots correspond to minimal, average and maximum relative importance of each individual attribute to the overall management success (OMS), ecological effectiveness, fishermen incomes and fishermen environmental commitment. Different colours indicate if the attribute has a significant (green), tentative (yellow) or negligible (red) role.

Using random forests^42^ and the Boruta add-on algorithm^43^ we highlight that the five most important attributes that significantly affect the OMS are: (1) *MPA enforcement*, (2) *fishermen engagement into MPA SSF management,* (3) *presence of fishermen within the management board*, (4) *presence of an incentive promoting sustainable fishing*, (5) the *existence of a management plan for SSF* (Fig. 2).

*HDI* (*Human Development Index*; a proxy for country development) also contributed significantly in determining OMS and was ranked as the sixth most important attribute. The average Relative Importance (RI) of *HDI* was closer to the two tentative attributes (i.e., the *portion of each MPA covered by no-take zone* and the *restriction of fishing rights exclusive*) than to the other five confirmed attributes. Likewise, *HDI* average RI was lower than the lowest RI of the first five attributes. Unlike the first five confirmed attributes, *HDI* cannot be altered by MPA management bodies to reach successful management of SSFs. Because *HDI* is beyond the control of management boards and its low RI, we do not include *HDI* in the pool of the key attributes that can increase marine protected area performance for small-scale fisheries management.

When focusing on the effect of the attributes on each outcome, it is clear that significant attributes differ depending on the focus outcome (Fig. 2), reinforcing the concept that fisheries and MPAs are complex SES^33^,^34^,^44^. Given this complexity, management must address problems related not only to the resources themselves, but also to the stakeholders targeting them.

**Figure 3:**
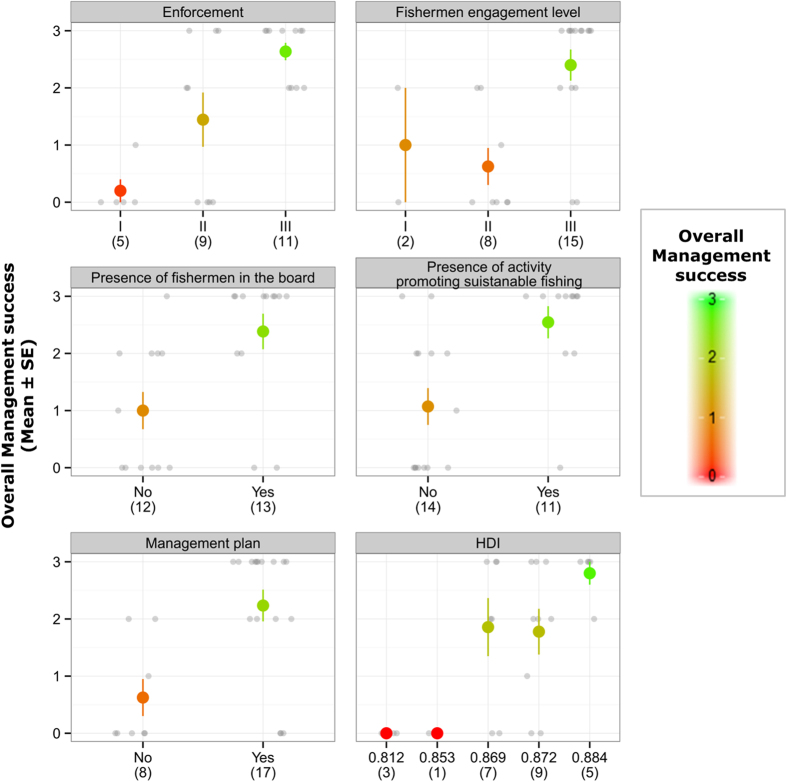
Effects of six significant attributes on overall management success (OMS). Average (±S.E.) OMS for each level of the six significant attributes. Colours denote average magnitude of success from red (null) to green (high). Sample size (number of MPAs; n) is provided under each level.

*MPA enforcement* was identified as the attribute with the highest RI. Specifically, OMS is higher in MPAs with high *enforcement* than in MPAs with low or medium *enforcement* (Fig. 3), with a similar pattern for each of the three single outcomes. It is generally well-known that the cessation of poaching because of high enforcement has ecological benefits^21^,^22^ because fish communities and populations are able to thrive. Nevertheless, the strong relationship between high *enforcement* and fishermen incomes, fishermen environmental commitment and OMS suggests an important role of *enforcement* not only in ecology, but also on economic and social aspects. These relationships suggest that the ecological benefits determined by high enforcement (i.e. increase in fish density/biomass within the MPA) most likely translate into economic benefits for fishermen via spill-over processes^26^ enhancing fisheries catches in the buffer zones. Subsequently, the perceived economic benefits by the fishermen contribute to increased compliance by the fishermen.

In the past, increasing the level of enforcement was demanding on the finances and resources of an MPA’s management body, especially when an MPA covers a large area. However achieving higher enforcement has become easier given the low cost of new technologies, such as unmanned aerial vehicles (i.e. conservation drones), and automatic ship identification systems (AIS)^45^. If MPAs are to combat the poaching and illegal fishing that accounts for a loss of US$10–23 billion^46^ globally, and a loss of more than 27000 jobs (i.e. 13% of total fisheries employment) in Europe^47^, there is a strong need for MPAs to develop strategies aimed at ensuring high enforcement.

*Fishermen engagement into MPA SSF management* was highlighted as the second most relevant (in terms of RI) attribute affecting OMS. Specifically, MPAs that actively engage fishermen within SSF management display a higher OMS than MPAs where fishermen have a marginal or passive role (Fig. 3). Likewise, the *presence of fishermen within the management board* (ranked as third most important attribute) is associated with successful SSF management. Our results agree with previous studies suggesting that participation of local users in the management of common pool resources is associated with positive outcomes^40^,^41^. In addition, we note the significant role of *fishermen engagement* in determining fishermen environmental commitment. This suggests that user participation in management can lead to perceived legitimacy of MPA management, a step crucial in determining user compliance^40^. Compliance can thus be related to a range of contextual conditions and processes, rather than just to the level of enforcement^44^. However, despite the positive influence of *fishermen engagement* in SSF management, only 60% of MPAs actively engage fishermen, and only 52% of MPAs examined had a fishermen representative on the management board (Supplementary Table S2).

In our study, compliance of local professional fishermen partially determines the measure of fishermen environmental commitment and OMS (see Supplementary methods). The significant correlation between fishermen environmental commitment and *enforcement*, and OMS and *enforcement*, can therefore be expected assuming that enforcement results in compliance. However, factors regulating compliance are highly complex^31^,^44^,^48^ with a limited effect of enforcement^44^,^49^, contradicting classical deterrence theory. These results suggest that if stakeholders are not engaged in the management process, more surveillance and enforcement could result in a negative attitude of stakeholders toward the MPA. This, in turn, would further distance MPA managers from stakeholders, potentially resulting in law infringements by local fishermen. In contrast, when fishermen are involved in management they feel that enforcement safeguards their rights, resulting in informal commitment of fishermen to sustainable SSF practices. In our case studies, formal and informal participation of fishermen in rulemaking and on MPA management boards likely leads to perceived legitimacy^50^ of SSF resulting in successful outcomes. Similar results have been seen in forestry where the participation of local forest users in forestry rulemaking resulted in higher tree species richness and increase in subsistence livelihoods^40^. However, understanding the causal mechanisms underlying the fisher-manager-enforcement relationship requires further work and goes beyond the scope of this paper. Nonetheless our findings clearly support a participatory and decentralised governance in fisheries^12^, and highlight, for the first time, that informal fishermen engagement in the Mediterranean can mimic the benefits of co-management seen in other regions^34^,^35^. This evidence is particularly relevant considering that in the Mediterranean, strong state–federal governance frameworks and institutional arrangements represent major barriers to formal power sharing between public authorities and stakeholders (i.e. co-management)^51^.

The *presence of SSF management plans* has a clear positive effect on OMS (Fig. 3). Despite this, 32% of the investigated MPAs did not have a SSF management plan (Supplementary Table S2) suggesting a need for wider implementation, and to develop plans that specifically address fisheries management with an emphasis on the participatory processes. Management plans can be formal or informal arrangements between MPA management body and fishermen, but they should detail the agreed objectives of the fishery and specify the management rules and regulations. Management plans should also contain a strategy aimed at promoting either traditional or novel mechanisms toward sustainable SSF. This is particularly relevant considering that MPAs that allow and promote sustainable SSF through labelling and awareness campaigns are associated with high OMS (Fig. 3). This highlights the potentially rewarding “marriage” between SSFs within MPAs and ecolabeling^52^, contradicting the widely criticised bias of sustainable fisheries initiatives against SSF^11^,^52^,^53^. This bias occurs because the development of eco-labels for SSF is generally limited by costs that are not manageable within SSF communities^11^,^52^. However, these costs could be reduced by capitalising on routine data collection, assessment and management activities already carried out in well-managed MPAs.

The low but statistically significant RI of *HDI* points out that successful management is more difficult to achieve in low HDI countries (Fig. 3). A lack of funding dedicated to MPAs most likely impedes effective enforcement while at the same time not allowing for programmes that engage fishermen into MPA SSF management, nor the ability to promote sustainable SSF. This drawback should be duly acknowledged given the high number of countries (both in the Mediterranean and globally) that fall within the low HDI range. Evidence within the literature concerning the role of HDI status affecting SES management is inconsistent. Some studies suggest a negligible role of HDI status^34^ while others indicate that high HDI values are associated with greater management success^41^. Reasoning behind this mismatch is complex and can be related to social and/or political situations, and are therefore difficult to generalise.

The results for the role of *portion of each MPA covered by no-take zone* and *only local fishermen allowed* (operated in 60% of the MPAs investigated in this study, see Supplementary Table S2) were inconclusive but their RI in determining OMS was similar to the value of HDI. Increasing the proportion of each MPA covered by a no-take zone could provide both ecological and socio-economic benefits. Larger no-take zones protect a larger portion of fish stocks and promote density dependent spill-over processes^26^ thus enhancing fisheries catches in the buffer zones^17^,^24^,^54^. On the other hand restrictions granting fishing rights exclusively to local fishermen could provide additional benefits and increase OMS. From this perspective combining Territorial Use Rights for Fisheries (TURFs) with MPAs could further increase the perceived legitimacy of management decisions by providing fishermen with “ownership” of local fisheries resources. Examples of benefits related to TURFs are available globally^55^, however this management tool remains largely under-utilised in the Mediterranean.

Interestingly, *leadership among fishermen* was not a determinant to successful management of SSF. This is in contrast with results from fisheries of other regions^34^ and also seems counterintuitive given the need for fishermen engagement. The presence of a leader among fishermen can potentially facilitate dialogue between fishermen and MPA management bodies, therefore enhancing fishermen engagement into SSF management. However, inconsistency in our results could be specific to MPAs where management bodies can catalyse the action of the fishermen community toward support in management, mimicking the role usually acted by fishermen leaders.

**Figure 4:**
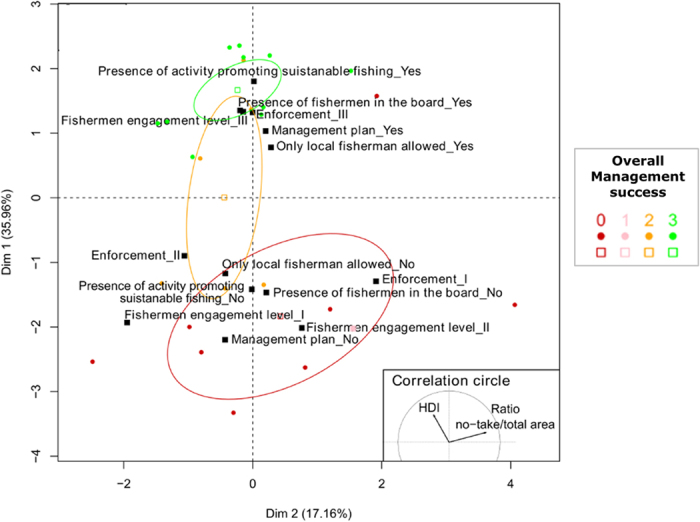
Factor Analysis of Mixed Data. The eight most important attributes (six significant and two tentative as detected by random forests and Boruta) detected for overall management success (OMS) were used as active variables. The OMS was added as a supplementary variable. Black filled squares indicate centroids of each factor levels. Coloured points indicate individual MPAs, colours indicating MPA success score. White-filled squares with coloured outlines indicate centroids of MPAs sharing the same OMS and ellipses indicate centroids 95% confidence intervals.

Highly successful MPAs (i.e. OMS = 3) show high similarity in the five most important attributes (as denoted by their clear grouping in Factor Analysis of Mixed Data; Fig. 4) with moderate correlations among four attributes (i.e. *fishermen engagement, presence of fishermen in management board, presence of a management plan* and *presence of activity promoting sustainable fisheries*) (Supplementary Figure S1). Conversely, MPAs with lower OMS have large variability in conditions determining their lack of success. This suggests that a wide range of conditions can result in unsuccessful/moderately successful SSF management in MPAs, while a very specific combination of circumstances can determine successful cases. Because success in our study cases was determined mainly by five shared attributes, and few studies had subsets of these five attributes, we are unable to determine if subsets would result in successful SSF-MPA management. However, the presence of these five attributes may be inherent of MPA management (e.g. promotion activities of sustainable fisheries is likely to be supported when a management plan is in place and fishermen are engaged) where good practices can facilitate the onset of other good practices.

To quantify tangible benefits delivered by successful MPAs, we collected extensive field data at one successful case study (Torre Guaceto MPA, Italy) by using underwater visual census and monitoring of SSF catch landings. At this MPA, four of the five key attributes have been implemented except for *presence of a fishermen representative* on the MPA management board. At Torre Guaceto the implementation of these four key attributes led to: a (i) 428% increase in total fish biomass in NTZ compared to external, unprotected areas, (ii) 128% increase in fishermen revenues when they operate within the MPA buffer zone compared to fishing outside the MPA, and (iii) dramatic increase in the commitment of local fishermen to environmental issues (i.e. the number of fines for illegal fishing after MPA establishment dropped to nearly zero after the implementation of the key features. Furthermore, the fishermen now participate in research and environmental programs).

Single-species management strategies generally employed for large-scale fisheries^7^ are not suited for the dynamic context of SSFs. However, alternative management strategies for SSF have been missing because successful examples were yet to be characterised. Here, we identify five key attributes that are suitable to manage SSFs. Significant economic and social commitments are required to implement the key attributes we have highlighted. Nevertheless, these commitments can be mitigated in part by implementing SSFs within an MPA framework. If successful, the benefits can be considerable for local coastal communities via increased revenue, they can achieve conservation goals and finally, they can maintain profitable exploitation of SSF resources. Products coming from successfully managed SSFs within MPAs would allow managers and policy-makers to satisfy the growing public demand for responsible seafood consumption^11^,^52^.

Although SSF-MPA systems are highly complex, the large range of socio-ecological conditions we examined make it likely that our key attributes could prove beneficial to SSF in other geographical locations in the Mediterranean Sea. Therefore, we can suggest that the allocation of some public expenditures from current fisheries subsidies (globally accounting for more than 30 billion US$/year^56^) to actions aimed at setting key attributes in MPAs (e.g. effective patrolling, stakeholders capacity building) will produce substantial ecological, economic and social benefits to society.

## Methods

### Database compilation

We use the term MPA *sensu lato* to define any marine area where human activities are restricted for conservation and/or management purposes, and that generally embed no-take zones into buffer zones. Furthermore, we made no distinction based on MPA legal status (e.g. national park, regional MPA, marine reserve etc.). However because a considerable part of the information gathered for each MPA had to be provided by MPA managers, we restricted our investigation to Mediterranean MPAs that have a management body. This criterion excluded a large proportion of MPAs belonging to the Natura 2000 network although many are in the process of establishing a management body. This lead to 153 potential MPAs^19^. From the pool of possible MPAs, we randomly selected 75, representing approximately half of all possible MPAs. Only half of the MPAs were selected because of the effort needed to contact each MPA management body (i.e. multiple direct contacts via e-mail and/or phone calls with MPAs’ managers). Also by selecting only half of the possible MPAs, we were able to maximise the data gathered for each MPA.

Information about each MPA was obtained through multiple sources. These included: (1) questionnaires emailed to MPAs managers and scientists, (2) review of international ISI scientific literature, (3) review of studies published on a national/local level and (4) review of grey literature and unpublished studies (e.g. project report) (see Supplementary Methods for a detailed description of data gathering procedure). Of the 75 randomly selected MPAs, 34 MPAs replied to the questionnaire. We therefore retained these 34 in our study while all the others were discarded due to a lack of critical information specific to the study.

At first we collected information on the largest number of attributes possible in order to thoroughly describe a range of differing management and social situations. However some attributes were removed because exhaustive data (e.g. about fishing effort, number of hours of surveillance, MPA funds for surveillance) could not be obtained or they had low relative discriminating power among the MPAs. For instance, when identifying the “multiple fishing gears allowed within the MPA”, the same score and/or category was attributable to more than 95% of the MPAs considered. This procedure resulted in 20 attributes being included in the study (Supplementary Table 2).

Outcomes were considered as three response variable. They were: (1) ecological effectiveness, measured as an increase in fish density or biomass as a result of the implementation of the MPA or when compared with open access areas, (2) fishermen incomes, measured as an income increase as a result of the implementation of the MPA or when compared with open access areas, and (3) fishermen environmental commitment, measured as their commitment to MPA SSF management practices and participation to research and environmental programmes. These three outcomes were defined on a binary scale denoting presence or absence (1 or 0 respectively) as done in Gutiérrez *et al*.^34^. This dichotomous coding scheme was chosen because the studies had differing techniques and sampling schemes, and a lack of temporal series concerning the number of fines. These problems also prevented the estimation of a response ratio for each of the three outcomes. However this coding schema is fully suitable to identify the attributes significantly contributing to successful management of SSF in MPAs that represented the aim of our study^34^.

Ecological effectiveness and fishermen incomes were based on a review of the scientific and grey literature as well as reports from the management bodies of the MPA. In a few cases the status was determined from our own unpublished results. Fishermen environmental commitment was determined by using information provided through questionnaires. For a detailed description of the rationale behind each outcome see the Supplementary Methods.

Information concerning at least two outcomes was missing from six MPAs and a further three forbid SSFs. These MPAs were removed from the analysis leading to a total of 25 MPAs being investigated. Based on a sensitivity analyses, reported in Supplementary Methods and Supplementary Table S3, we assessed that our sample size (i.e. n = the number of marine protected areas included in the present study) represented a replication level adequate to provide reliable estimation of the relevance of the attributes considered in determining overall success (see Supplementary Methods and Supplementary Table S3).

Finally, data were compiled for 23 variables (20 attributes and 3 outcomes) for each of the 25 MPAs where SSF were allowed within their boundaries and for which evidences about at least 2 of the 3 outcomes were available.

To assess the potential effect on our results of misreporting related to outcome and/or oversights of useful literature, we performed a sensitivity analysis. This analysis showed that our data were highly robust to moderate miscoding of the three outcomes (see Supplementary Methods, Supplementary Table S4 and Supplementary Figures S7–8).

### Statistical analyses

This data matrix contained 575 cells of which 3.6% of the attributes and 6.7% of the outcomes contained missing data. The missing data were compensated via missForest^57^, an iterative imputation method based on a random forest that can successfully impute missing values. The method uses multicollinearity of surrounding cells, thus data were imputed separately for attributes and outcomes. This prevented circular reasoning and the introduction of spurious relationship between attributes and outcomes.

The overall management success score (OMS) was computed including the imputed (missForest) outcomes. We assessed the strength and significance of the correlative relationships between the OMS, the 3 outcomes and the 20 attributes (i.e. predictors) by using random forests^42^ and the Boruta add-on algorithm^43^. The random forest method was suitable for our dataset because it can cope with small sample sizes, a large number of predictors, complex interactions and highly correlated quantitative and/or qualitative attributes^42^. The strength of correlative relationships between the outcomes and each attribute were indicated by the relative importance of each attribute to the predictive accuracy of the random forest. The significance of these relationships was assessed with the Boruta algorithm. The Boruta algorithm tests the significance and predictive accuracy of each attribute by comparing the observed score against a set the randomly permuted attributes (500 permutations across objects). Hence, this provides inference about the attribute importance, which may be either confirmed (importance higher than random) or rejected (importance lower than random probes), although in some cases the attribute may be judged neither confirmed nor rejected and thus finally marked as tentative^43^. The set of relevant attributes may contain correlated and redundant variables. Also, the correlation of the attribute with the outcomes does not imply causative relation; it may arise when both are independently correlated with a third variable.

The random forest algorithm implemented in the R package randomForest^58^ has three hyperparameters known to affect RF model predictive accuracy and attribute importance estimates: (1) ntree, the number of trees grown, (2) mtry, the number of attribute randomly selected when growing one tree, and (3) nodesize, the minimum size of terminal nodes. Therefore it is crucial to tune the three hyperparameters in order to optimise the RF model. To do so we followed the procedure described in Supplementary Methods and Supplementary Figures S2–S5.

Once the random forests were optimised and the significant, relevant, attributes were identified we assessed collinearity among the eight most relevant attributes by using Kendall’s rank correlation coeffecient^59^. P-values under the null hypothesis of no association were obtained by normal approximation with continuity correction^59^.

In order to characterise the multivariate relationships among the eight attributes identified as important (i.e. the ones detected as significant and tentative) by Boruta (in part collinear, Supplementary Figure S1), we carried out a Factor Analysis of Mixed Data (FAMD) by using the R package FactoMineR^60^. The eight most important attributes detected by Boruta for OMS were used as active variables. The success score was added as a supplementary variable in order to appreciate the directionality of attributes effects.

## Additional Information

**How to cite this article**: Di Franco, A. *et al*. Five key attributes can increase marine protected areas performance for small-scale fisheries management. *Sci. Rep.* **6**, 38135; doi: 10.1038/srep38135 (2016).

**Publisher's note:** Springer Nature remains neutral with regard to jurisdictional claims in published maps and institutional affiliations.

## Supplementary Material

Supplementary Information

## Figures and Tables

**Figure 1:**
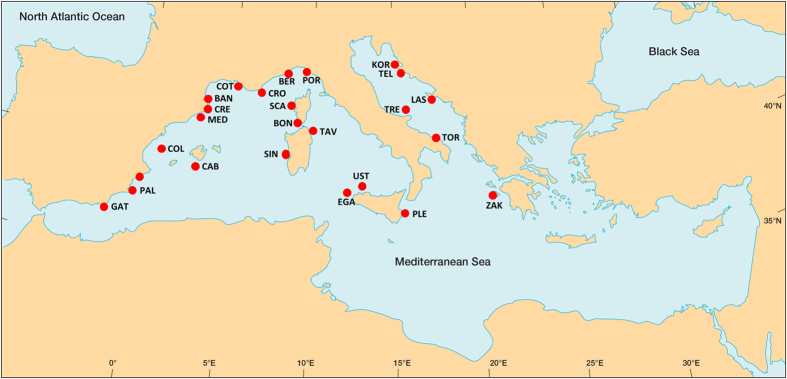
Location of the marine protected areas (MPAs) included in the present study. BAN = Banyuls, BER = Bergeggi, BON = Bonifacio, CAB = Cabrera, COL = Columbretes, COT = Côte Bleue, CRE = Cap de Creus, CRO = Port Cros, EGA = Egadi, GAT = Cabo de Gata, KOR = Kornati, LAS = Lastovo, MED = Medes, PAL = Cabo de Palos, PLE = Plemmirio, POR = Portofino, SCA = Scandola, SIN = Penisola del Sinis, TAV = Tavolara, TEL = Telašćica, TOR = Torre Guaceto, TRE = Tremiti, UST = Ustica, ZAK = Zakynthos. For details on each MPA see Supplementary Table S1. Map was generated using the open source QGis software version 2.6.1 (http://www.qgis.org/en/site/).
