# Supplementary material for: Five key attributes can increase marine protected areas performance for small-scale fisheries management
Source: Sci Rep. 2016 Dec 1;6:38135. doi: 10.1038/srep38135 (PMC5131471; doi:10.1038/srep38135)
Supplement: Supplementary Information [file srep38135-s1.pdf]

Five key attributes can increase marine protected areas performance for small-scale fisheries management

Antonio Di Franco, Pierre Thiriet, Giuseppe Di Carlo, Charalampos Dimitriadis, Patrice Francour, Nicolas Gutiérrez, Alain Jeudy de Grissac, Drosos Koutsoubas, Marco Milazzo, María del Mar Otero, Catherine Piante, Jeremiah Plass-Johnson, Susana Sainz-Trapaga, Luca Santarossa, Sergi Tudela and Paolo Guidetti

Supplementary Information

Supplementary methods

Data gathering procedure

In order to compile information for each MPA, we used multiple sources: 1) first we e-mailed questionnaires designed to obtain specific data from each MPA on management of SSF (e.g. the number of boats allowed to fishing in the MPA, gears allowed, regulation, involvement of fishermen in the management process) to MPAs managers and scientists. Within the questionnaire we used forty-eight questions to collect information about SSF management in each MPA, together with the related ecological and socio-economic effectiveness evidences. In this way, managers/scientists provided us with information that we would otherwise not have been able to get, compiling an unprecedented dataset for the Mediterranean. Direct contacts via e-mail and/or phone calls with MPAs' managers and scientists has been also carried out to refine responses from questionnaires. Due to the very patchy nature of small-scale fishermen communities in the Mediterranean Sea and the general lack of associations (and therefore representatives) grouping fishermen at a small spatial scale (e.g. fishermen operating within and/or close to an MPA) we were not able, on such a large scale, to interview fishermen about the issues investigated. In that perspective it could be argued that we collected information from a narrow range of informant types (i.e. MPA managers and scientists), however it has to be stressed that in the questionnaire we did not ask for respondents' perception but only for official and factual information (e.g. number of fines, management procedures adopted to engage fishers into management). In addition, for what concerns the level of fishermen engagement into SSF management, a variable that could be prone to perception bias, we performed fact-checking in a subset of MPAs where we have long-lasting collaboration with both managers and small-scale fishermen (i.e. Torre Guaceto, Scandola,

Tavolara, Portofino). In all the 4 case studies we detected a match between information provided by managers and both fishermen perspective and our perception on the field. We can therefore reasonably consider that our database is free from bias related to partial perceptions, and all the issue related to different perspective among stakeholders go beyond the scope of the present work.

2) we also reviewed scientific literature obtained through a comprehensive search of various electronic library databases (Web of Science, Google Scholar, Scopus) using key words (different combinations of: fish*, fisher*, CPUE, catch*, small scale, coastal, artisanal, manag*, outcome*, benefit*, socio*, econom*, and income*) and the name of each MPA and following up references therein, with an additional search performed on Google;

We expanded our review to 3) studies published on a national/local level, and on conference proceedings. As such studies are often unavailable on-line and unreported in scientific databases, we searched these sources in our personal archives or explicitly asked colleagues working in other scientific institutions, NGOs, MPAs, etc., and to 4) grey literature, unpublished studies (e.g. project report) carried out by the MPAs' management bodies and directly provided by the MPAs. This procedure also allowed us to account for other documents unavailable from more conventional sources.

Manuscripts in different languages (i.e., English, Spanish, French, Italian and Croatian) were reviewed and processed. Literature search was performed in October 2014. Papers and other documents published later than this date were not considered in our analysis.

Thirty-four MPAs replied to the questionnaire and were therefore retained in our database. All the other MPAs were discarded due to lack of crucial information.

Data gathering procedure

For both ecological effectiveness and increase in fishermen incomes, positive effect of an MPA was related to a score of 1 (presence). Absence or evidence of positive effects of MPAs were assessed as a result of the implementation of management (before-after analysis) or when comparisons were available between MPAs and surrounding unprotected areas (control-impact analysis). When information about fishermen incomes was missing we used CPUE or CPUA (catch per unit of effort or area, depending on information availability from each MPA) as a proxy of fishery benefits (higher catches or incomes) assuming as constant the 'fish price' between each MPA and its unprotected "control" area (or along the time), and assuming the fishermen incomes related to the amount of fish collected. In this study, we have also assumed the cost of fishing as constant between each MPA and its unprotected "control" area (or across time). These assumptions make thus possible to provide a simplified 'fishing income' estimate, as this latter is related not only to the amount of fish collected, but also to a number of other variables, like e.g. the fishing operational costs (such as fuel, personnel), the specific techniques used and the location of fishing areas. Revenues are related also to species composition (being some species much more valuable than others) and fish size (as bigger fish are more valuable than small ones). However, as a general rule, the most valuable species are the ones that are most targeted by fishing and also the ones more benefiting (in terms of increased density and size) from MPA protection[36,38,55]. From this perspective, an equal-weight catch coming from an ecologically effective MPA (composed by more commercially valuable fish of larger size) could be more valuable than a catch coming from an unprotected area. Accordingly, in this study the positive effect of an MPA on fishermen incomes could be underestimated, but the general lack of detailed data makes it impossible to provide more in-depth analyses on fishermen incomes in MPAs.

Fishermen environmental commitment was defined by looking at 1) compliance with MPA rules considering if authorized fishermen to operate within the MPA boundaries committed infractions for illegal fishing, and 2) fishermen participation in research/environmental programs developed in the local MPAs considering if multiple fishermen systematically participated to these activities. If fishermen did not committed any infractions and were engaged in research/environmental programs a score of 1 was assigned to the MPA.

Fishermen environmental commitment was identified as an outcome in order to capture the social dimension of the complex socio-ecological system represented by SSF management in MPAs and to include it in the building of the overall success score. This outcome is a proxy of fishermen commitment toward MPA management goals and, on the other hand, of social conflicts/friction of fishermen versus the management body (that represent a concrete hurdle to fishery management in MPAs[19]); in this perspective a successful management should target to reduce/annul these conflicts and foster fishermen commitment in the same way as it should target economic benefits for fishermen and ecological benefits in terms of increased fish density/biomass.

Random forests optimization

The random forest algorithm implemented in the R package randomForest[58] has notably 3 hyperparameters known to affect RF model predictive accuracy and attribute importance estimates: 1) ntree, the number of trees grown, 2) mtry, the number of attribute randomly selected when growing one tree, and 3) nodesize, the minimum size of terminal nodes. Therefore it is crucial to tune the 3 hyperparameters in order to optimize the RF model. To do so we adopted the following steps: 1) assess the minimal ntree ($ntree_m$) so that the out-of bag error-rate stabilize at low value using default mtry and nodesize (i.e. 4 and 1 respectively) and select an ntree ($ntree_o$) higher that $ntree_m$ because this will not cause RF to overfit[33]; 2) find the best combination of the hyperparameters mtry and nodesize (i.e. the combination that minimizes the mean error-rate) by using a grid search of 9 x 10 combinations of hyperparameters (i.e. the mtry values tested were from 1 to 9, the nodesize values tested were from 1 to 10) by fitting 100 RF for each combination of hyperparameters and using the mean out-of bag error-rate over the 100 runs; 3) check if the $ntree_o$ selected after step 1 (i.e. ntree=1000) is still large enough to stabilize the out-of bag error-rate when using the best combination of mtry and nodesize found in step 2. These steps were run for each of the 4 outcomes (i.e. OMS, ecological effectiveness, fishermen incomes, fishermen compliance and commitment) separately (see Supplementary Fig. S2-S5 for details about the 3 steps for each of the outcomes).

In order to perform an additional check of the sensitivity of Boruta outputs to different hyperparameters selection (i.e. fine tuning in mtry and nodesize selection), we qualitatively compared outputs of Boruta models fitted with the 3 best combinations of hyperparameters identified by using the above mentioned grids search.

For none of the 4 outcomes considered we detected a relevant variability among the outputs of the random forests models with the 3 best combinations of hyperparameters (Supplementary Fig. 6),

with this finding highlighting the robustness of RF and Boruta to fine tuning in hyperparameters selection.

Random forests and Boruta sensitivity to the number of MPAs considered

In order to assess if our sample size (i.e. n = the number of marine protected areas included in the present study) represented a replication level adequate to provide reliable estimation of the relevance of the attributes considered in determining overall success, we assessed the sensitivity of the RF model to the n considered. Specifically we implemented in R a script to repeat 1000 times (independently) random sampling without replacement of n_i MPAs (a subset of all the 25 MPAs considered, with $15 \leq n_i \leq 25$), and compute Boruta algorithm using ntree=1000, mtry=2 and nodesize=7, (i.e. the best combination of hyperparameters found for the whole dataset). For each Boruta run, the decision relative to each attribute (confirmed, tentative, or rejected) was stored. Finally for each attribute at each n_i, the proportion of Boruta decision over the 1000 repetitions was computed (Supplementary Fig. 7).

We show that for the 5 most relevant attributes in determining overall success identified in the main study (i.e. MPA enforcement, fishermen engagement, presence of a management plan, presence of fishermen in the MPA board, presence of activity promoting sustainable fishing) 22 MPAs among the ones investigated represent an adequate level of replication, because 100% of Boruta decisions are consistent with the Boruta decision performed over the whole dataset (i.e. containing the 25 MPAs). Lower n are adequate for fishermen engagement level, presence of fishermen in the board and Enforcement level (Supplementary table 3).

Random forests and Boruta sensitivity to possible miscoding in MPA success score estimation

We assessed the sensitivity of Boruta outputs to potential miscoding of the three outcomes (ecological effectiveness, fishermen incomes and fishermen environmental commitment) used to build the overall success score. Miscoding could be due to incorrect information reported in the documents (i.e. papers/reports) we used to code each variable for each MPA, or to involuntary exclusion of useful reports/papers particularly difficult to find through our bibliographic search and unknown by MPA managers. Despite high unlikely (due to our extensive literature search and the large use of peer-reviewed papers) this potential bias cannot be excluded. In this perspective we implemented in R a script to repeat 1000 times (independently) random forests and Boruta with the response variable (i.e. overall success score) modified by randomly summing or subtracting 1 at an increasing number of MPAs (n_i) in our dataset. We used ntree=1000, mtry=2 and nodesize=7, (i.e. the best combination of hyperparameters found for the whole dataset). For each Boruta run, the decision relative to each attribute (confirmed, tentative, or rejected) was stored.

Finally for each attribute at each n_i, the proportion of Boruta decision over the 1000 repetitions was computed (Supplementary Fig. 8).

Boruta outputs were proven to be highly robust to moderate variation in overall success score (i.e. summing or subtracting 1) especially for three attributes: enforcement, level of fishermen engagement and presence of a management plan. For this attributes an outcome should have been miscoded in at least 6-10 (depending on the attributes, see Supplementary Table 4) in order to obtain Boruta outputs deviating from the ones we obtained in the present study.

We assessed also the effect of potential more drastic miscoding by implementing a script similar to the one described above but modifying the overall success score by randomly summing or subtracting 1 or 2 at an increasing number of MPAs (Supplementary Fig. 8).Also in this case the output was robust for 5 of the 6 significant attributes, and especially for enforcement, level of fishermen engagement and presence of a management plan (Supplementary Table 4). The outputs

of these analyses highlight that our results are highly robust to moderate miscoding and are robust even to more drastic miscoding.

Supplementary tables

Table S1. Summary information of case studies for which information was compiled (*n* = 34). Highlighted in grey are the definitive cases included in the final model (n = 25). NA: not assessed.

**MPA**	**Country**	**MPA size (in km^2)**	**MPA implementation year**	**OMS (overall management success)**	**References**
Alboran	Spain	21	1997	NA	
Banyuls	France	7	1974	2	1-5
Bergeggi	Italy	2	2007	2	6,7
Bonifacio	France	803	1999	3	1,8,9
Brijuni	Croatia	26	1983	NA	
Cabo de Gata	Spain	46	1995	0	10
Cabo de Palos	Spain	19	1995	3	2,11-15
Cabrera	Spain	87	1991	2	2,9,16,17
Cap de Creus	Spain	31	1998	0	18-21
Columbretes	Spain	55	1990	3	2,22-25
Côte Bleue	France	102	1983	3	1-3,26-30
Egadi Islands	Italy	540	1991	0	31
Gokceada	Turkey	10	1999	NA	
Habibas	Algeria	27	2003	NA	
Kas-Kekova	Turkey	166	1990	NA	
Kornati	Croatia	217	1980	0	32-34
Lastovo	Croatia	143	2006	0	35,36
Levante de Mallorca	Spain	113	2007	NA	
Medes	Spain	5	2001	3	2,3,16,37,38
Miramare	Italy	1	1986	NA	

Penisola del Sinis	Italy	267	1997	0	13,31,39,40
Plemmirio	Italy	25	2004	2	40-42
Port-Cros	France	271	1963	3	43-48
Portofino	Italy	3	1998	3	7,16,31,49,50
Scandola	France	10	1975	3	52-54
Tabarca	Spain	18	1986	2	1-3,55
Tavolara	Italy	159	1997	2	16,51,56-59
Telascica	Croatia	70	1988	0	60,61
Tor Paterno	Italy	14	2000	NA	
Torre del Cerrano	Italy	34	2009	NA	
Torre Guaceto	Italy	23	1991	3	16,51,62-64
Tremiti	Italy	15	1989	1	31,65
Ustica	Italy	161	1986	3	66-67
Zakynthos	Greece	83	1990	0	68

References

1. Forcada, A. *et al*. Effects of habitat on spillover from marine protected areas to artisanal fisheries. *Mar Ecol Prog Ser.* **379**: 197-211 (2009).

2. Goñi, R. *et al*. Spillover from six western Mediterranean marine protected areas: Evidence from artisanal fisheries. *Mar. Ecol. Prog. Ser.* **366**: 159-174 (2008).

3. Harmelin-Vivien, M. *et al*. Gradients of abundance and biomass across reserve boundaries in six Mediterranean marine protected areas: evidence of fish spillover? *Biol. Conserv.* **141**: 1829–1839 (2008).

4. Lenfant, P., Caro, A., Neveu, R. & Jarraya, M. Les débarquements de la pêche artisanale: de Leucate à Port-Vendres (2011). Rapport CEFREM pour Agence des Aires Marines Protégées (2012).

5. Lenfant, P. *et al*. Suivi temporel du peuplement ichtyque au sein et à proximité de la Réserve Naturelle Marine de Cerbère-Banyuls – Année 1 & 2 été – automne 2007 & 2009. Contrat Conseil Général des Pyrénées Orientales & CEFREM UMR 5110 CNRS-UPVD (2012).

6. Guidetti, P., Di Franco, A. & Bussotti, S. Valutazione della fauna ittica costiera e monitoraggio dell'effetto riserva presso l'Area Marina Protetta 'Isola di Bergeggi'. Report (2014).

7. Cattaneo-Vietti, R., Guidetti, P., Di Lorenzo, M. & Bussotti, S. Pre-valutazione dell'Effetto Riserva presso i cinque parchi marini della Liguria - Annualità 2010. Report (2011).

8. Albouy, C., Mouillot, D., Rocklin, D., Culioli, J.M. & Le Loch, F. Simulation of the combined effects of artisanal and recreational fisheries on a Mediterranean MPA ecosystem using a trophic model. *Mar. Ecol. Prog. Ser.* **412**: 207-221 (2010).

9. Office de l'environnement de la Corse. Synthese des resultats de 20 ans de collaboration entre l'office de l'environnement de la Corse et la Prud'hommie des Bouches de Bonifacio. Rapport de synthèse (2012).

10. Villamor, A. & Becerro, M.A. Species, trophic, and functional diversity in marine protected and non-protected areas. *J. Sea Res.* **73**: 109–116 (2012).

11. Esparza Alaminos, O. Estudio de la pesca artesanal en el entorno de la reserva marina de Cabo de Palos-Islas Hormigas : estrategias, efecto de la protección y propuestas para la gestión. PhD Thesis, University of Murcia (2010).

12. Lorenzi, M. Efectos a largo plazo de la reserva marina de Cabo de Palos – Islas Hormigas (Murcia, España) sobre la pesca artisanal. MSc Thesis, University of Murcia (2013).

13. Roncin. N. *et al*. Uses of ecosystem services provided by MPAs: how much do they impact the local economy? A southern Europe perspective. *J. Nat. Conser.* **16**: 256—270 (2008).

14. Hackradt, C.W. *et al*. Response of rocky reef top predators (Serranidae:Epinephelinae) in and around Marine Protected Areas in the western Mediterranean Sea. *PLoS ONE* **9(6)**: e98206 (2014). doi:10.1371/journal.pone.0098206

15. García-Charton, J. *et al*. Estudio de seguimiento de la Reserva Marina de Cabo de Palos – Islas Hormigas. Universidad de Murcia y Consejería de Agricultura, Agua y Medio Ambiente de la Comunidad Autónoma de la Región de Murcia. Report (2013).

16. Sala, E. *et al*. The structure of Mediterranean rocky reef ecosystems across environmental and human gradients, and conservation implications. *PLoS ONE* **7**, e32742 (2012).

17. Perez, C.C. Alternatives for the sustainable management of artisanal fisheries for crustaceans of commercial interest in Cabrera Archipelago National Park (Balearic Islands, Western Mediterranean). Bachelor's Degree in Environmental Science, University of Barcelona (2013).

18. Munõz, M., Lloret, J. & Vila, S. Effects of artisanal fisheries on the scorpaenids (*Scorpaena* spp.) reproduction in the marine protected area of Cap de Creus (NW Mediterranean). *Fish. Res.* **138**: 146-151 (2013).

19. Gómez, S., Lloret, J. & Riera, V. The decline of the artisanal fisheries in Mediterranean coastal areas: the case of Cap de Creus (Cape Creus). *Coast. Manag.* **34**: 217-232 (2006).

20. Lloret, J. & Riera, V. Evolution of a Mediterranean coastal zone: human impacts on the marine environment of Cape Creus. *Env. Manag.* **42**: 977-988 (2008).

21. Lloret, J., Casadevall, M. & Muñoz, M. Seguiment de la pesca artesanal al Parc Natural del Cap de Creus: Estudis 2008-2010. Generalitat de Catalunya. Departament de Medi Ambient i Habitatge. Parc Natural del Cap de Creus. Report (2010).

22. Goñi, R., Quetglas, A. & Renones, O. Spillover of spiny lobsters *Palinurus elephas* from a marine reserve to an adjoining fishery. *Mar. Ecol. Prog. Ser.* **306**: 207–219 (2006).

23. Goñi, R., Hilborn, R., Díaz, D., Mallol, S. & Adlerstein, S. Net contribution of spillover from a marine reserve to fishery catches. *Mar. Ecol. Prog. Ser.* **400**: 233-243 (2010).

24. Stobart, B. *et al.* Long-term and spillover effects of a marine protected area on an exploited fish community. *Mar. Ecol. Prog. Ser.* **384**: 47–60 (2009).

25. Díaz, D., Mallol, S., Parma, A.M. & Goñi, R. Decadal trend in lobster reproductive output from a temperate marine protected area. *Mar. Ecol. Prog. Ser.* **433**: 149–157 (2011).

26. Charbonnel, E., Leleu, K. & Bachet, F. Bilan des suivis de la pêche professionnelle et récréative dans le Parc Marin de la Côte Bleue. Rapport Parc Marin de la Côte Bleue, Fr (2013).

27. Leleu, K. Suivi et évaluation de la pêche professionnelle au sein d'une Aire Marine Protégée : protocoles d'enquêtes et indicateurs de pression et d'impact. Application au Parc Marin de la Côte Bleue. PhD Thesis, Université d'Aix-Marseille (2012).

28. Leleu, K. *et al.* Fisher's perceptions as indicators of the performance of Marine Protected Areas (MPAs). *Mar. Policy* **36**: 414-422 (2012).

29. Leleu, K. *et al.* Métiers, effort and catches of a Mediterranean small-scale coastal fishery: the case of the Côte Bleue Marine Park. *Fish. Res.* **154**: 93–101 (2014).

30. Bellier, E. *et al.* Marine reserve spillover: Modelling from multiple data sources. *Ecol. Inform.* **18**: 188–193 (2013).

31. Guidetti, P. *et al*. Italian marine protected area effectiveness: does enforcement matter? *Biol. Cons.* **141**: 699–709 (2008).

32. Markov-Podvinski, M. Recent state and seasonal fluctuations of nearshore settlements of fish, cephalopods and crustaceans in Kornati National Park. PhD thesis, University of Zagreb, Croatia. 220 pp. (In Croatian). (2011).

33. Staglicic, N. Assessing the effectiveness of marine protected areas in the eastern Adriatic. PhD thesis, University of Split and Dubrovnik, Croatia. 198 pp. (In Croatian). (2013).

34. Kruschel, C., Schultz, S.T., Bakran-Petricioli, T. & Petricioli, D. Comparing predator abundance and fish diversity in MPA sites (Kornati NP, Croatia) and adjacent sites exploited by fisheries. *Croatian Journal of Fisheries* **70, Supplement 1**: S35-S49 (2012).

35. Matić Skoko, S., Staglicic, N., Pallaoro, A., Vrbatović, A. & Bušelić, I. Zdravi pristup zdravoj hrani iz zdravog mora: valoriziranje ribe koja potječe iz zaštićenog područja. *Croatian Journal of Fisheries* **70 Supplement 1**: S39-S52 (2012).

36. Stagličić, N. Assessing the effectiveness of rotational protecion regime for the renewal and preservation of coastal fisheries resources in Nature Park Lastovsko otočje. Years 2010 - 2013. Institute of Oceanography and Fisheries, Split, Croatia. 50 pp. Report (2013).

37. Martin, P., Maynou, F., Stelzenmüller, V. & Sacanell, M. A small-scale fishery near a rocky littoral marine reserve in the northwestern Mediterranean (Medes Islands) after two decades of fishing prohibition. *Sci. Mar.* **76**: 607-618 (2012).

38. Stelzenmüller, V., Maynou, F. & Martin, P. Spatial assessment of benefits of a coastal Mediterranean Marine Protected Area. *Biol. Cons.* **136(4)**: 571-583 (2007).

39. Guidetti, P., Bussotti, S., Massaro, G. & Brundu, R. Indagine sulla piccola pesca professionale presso l'AMP Penisola del Sinis-Isola Mal di Ventre – seconda annualità. Project report (2012).

40. Nasti, A. & Marino, D. Aree Marine Protette e pesca: alla ricerca della governance. In Vallarola et al. Aree marine protette e pesca: alla ricerca delle buone pratiche condivise. Conference proceedings (2008).

41. Consoli, P. *et al*. The effects of protection measures on fish assemblage in the Plemmirio marine reserve (Central Mediterranean Sea, Italy): A first assessment 5 years after its establishment. *J. Sea Res.* **79**: 20–26 (2013).

42. Galfo, F., Pasolli, L. & Nicastro, A. Determinazione dell'Effetto Riserva indotto dalla gestione dell'Area Marina Protetta sulle popolazioni ittiche in relazione alle attività di pesca ed alle condizioni socio-economiche. Report (2014).

43. Bonhomme, P. *et al*. Suivi de l'effort de pêche professionnelle dans les eaux du Parc national de Port-Cros. Année 2012. Partenariat Parc national de Port-Cros & GIS Posidonie (2012).

44. Cadiou, G, Boudouresque, C.F., Bonhomme, P. & Le Diréach, L. The management of artisanal fishing within the Marine Protected Area of the Port-Cros National Park (northwest Mediterranean Sea): a success story? *ICES J. Mar. Sci.* **66**: 41–49 (2009).

45. Francour, P., Bodilis, P. & Cottalorda, J.M. Suivi des peuplements de poissons dans le cadre de l'Observatoire opérationnel de la Biodiversité du Parc national de Port-Cros. Contrat Parc National de Port-Cros et ECOMERS. Université de Nice-Sophia Antipolis et ECOMERS (2013).

46. Groupe d'Etudes du Mérou (GEM). Programme de recensement de la population du mérou brun (*Epinephelus marginatus*) du Parc national de Port-Cros 2008-2010. Convention GEM & Parc National de Port-Cros, Convention n°08-030 (2010).

47. Valls, A., Gascuel, D., Guénette, S. & Francour, P. Modeling trophic interactions to assess the potential effects of a marine protected area: case study in the NW Mediterranean Sea. *Mar. Ecol. Prog. Ser.* **456**: 201-214 (2012).

48. Colléter, M. *et al*. Fishing inside or outside? A case studies analysis of potential spillover effect from marine protected areas, using food web models. *J. Mar. Syst.* **139**: 383–395 (2014).

49. Molinari, A., Bava, S. & Tunesi, L. Valutazione dell'efficacia delle aree marine protette: spill-over e possibili effetti sulla pesca. Relazione sintetica intermedia sui risultati del primo anno di attività presso l'area marina protetta di Portofino. Project report (2005).

50. Cappanera, V. La pesca artigianale costiera: l'impatto della pesca sulla frazione ittica della fauna marina e sulle biocenosi del coralligeno. Report (2014).

51. Guidetti, P. *et al*. Large-Scale Assessment of Mediterranean Marine Protected Areas effects on fish assemblages. *PLoS ONE* **9(4)**: e91841 (2014). doi:10.1371/journal.pone.0091841

52. Le Direach, L., Ourgaud, M., Goujard, A. & Bonhomme, P. Suivi de l'effort de pêche professionnelle dans la réserve naturelle de Scandola (Corse). Données 2012. Contrat Parc naturel Regional de Corse & GIS Posidonie publ., Fr (2013).

53. Seytre, C. & Francour, P. A long term survey of *Posidonia oceanica* fish assemblages in a Mediterranean marine protected area: emphasis on stability and no-take area effectiveness. *Mar. Freshw. Res.* **65(3)**: 244-254 (2013).

54. Francour, P., Bodilis, P. & Cottalorda, J.M. Evaluation des peuplements de poissons dans la Réserve Naturelle de Scandola et à proximité de ses limites : les apports de la méthode FAST. Contrat Parc Naturel Régional de Corse et ECOMERS. Université Nice-Sophia Antipolis et ECOMERS, publ., Nice (2011).

55. Forcada, A., Valle, C., Sánchez-Lizaso, J.L., Bayle-Sempere, J.T. & Corsi, F. Structure and spatio-temporal dynamics of artisanal fisheries around a Mediterranean marine protected area. *ICES J. Mar. Sci.* **67**: 191–203 (2010).

56. Di Franco, A., Bussotti, S., Navone, A., Panzalis, P. & Guidetti, P. Effects of total and partial restrictions to fishing on Mediterranean rocky-reef fish assemblages. *Mar. Ecol. Prog. Ser.* **387**: 275-285 (2009).

57. Pitzianti, G.M., Cattaneo Vietti, R. & Panzalis, P.A. Monitoraggio della pesca professionale nell'Area Marina Protetta di Tavolara - Punta Coda Cavallo. Project report (2011=.

58. Sahyoun, R. *et al*. Protection effects on Mediterranean fish assemblages associated with different rocky habitats. *J. Mar. Biol. Assoc. U.K.* **93(2)**: 425–435 (2013).

59. Guidetti, P. *et al*. Relazione tra 'effetto riserva' e piccola pesca nell'ottica della sostenibilità all'interno dell'Area Marina Protetta 'Tavolara-Punta Coda Cavallo'. Report (2014).

60. Stagličić, N. Assessment and monitoring of the state of coastal fisheries resources for Nature Park Telašćica. Years 2011 - 2013. Institute of Oceanography and Fisheries, Split, Croatia. Report (2013).

61. Matić-Skoko, S., Stagličić, N. & Pallaoro ,A. Coastal fish community inventarisation and assessment of coastal fisheries resources of Nature Park Telašćica (2011).

62. Di Franco, A. *et al*. Assessing Dispersal Patterns of Fish Propagules from an effective Mediterranean Marine Protected Area. *PLoS ONE* **7(12)**: e52108 (2012).

63. Guidetti, P. & Claudet, J. Comanagement practices enhance fisheries in Marine Protected Areas. *Cons. Biol.* **24**: 312–318 (2010).

64. Guidetti, P., Bussotti, S., Pizzolante, F. & Ciccolella, A. Assessing the potential of an artisanal fishing co-management in the Marine Protected Area of Torre Guaceto (southern Adriatic Sea, SE Italy). *Fish. Res.* **101**: 180–187 (2010).

65. Guidetti, P., Bussotti, S. & Di Franco, A. Monitoraggio della fauna ittica presso l'AMP Isole Tremiti ai fini della valutazione degli effetti della protezione/gestione e regolamentazione della pesca. Project report (2013).

66. Chemello, R., Milazzo, M., Palmeri, A., Todaro, A. & Gorgone, R. Valutazione dell'efficacia delle aree marine protette: spillover e possibili effetti sulla pesca. Report (2004).

67. Di Franco, A. unpublished data

68. Dimitriadis, C. et al. Management Measures for Fisheries in the Marine Protected Area of the National Marine Park of Zakynthos. MedPAN North Project, Final report (2013).

Table S2. Variables included in the study in order to describe the interdisciplinary features of small-scale fisheries management. Each variable is identified as attribute (predictive variable) or outcome (response variable). Name, description, coding of each variable is provided. Depending on the nature of each variable (binary, categorical or continuous) proportion of MPAs with each attribute/outcome, median or mean (±SE) values, respectively, for the 25 MPAs retained in the analysis is provided.

**Attribute/outcome**	**Name**	**Description**	**Coding**	**Frequency or median or mean** (±SE)
Attribute	Fishermen predominantly organized in associations	The majority of fishermen operating within the MPA are member of a unique fishermen association	Binary: Yes(1)/No(0)	Frequency: 92%
Attribute	Fishermen financially compensated	The MPA provides a financial compensation to fishermen participating in scientific surveys and data collection within the MPA	Binary: Yes(1)/No(0)	Frequency: 60%
Attribute	Fishermen practicing fishing tourism	Fishermen regularly fishing within the MPA perform fishing tourism as a side activity	Binary: Yes(1)/No(0)	Frequency: 36%
Attribute	HDI	Human Development Index. Compounded index of "human development" (life expectancy, literacy rate, GDP), considered as proxy for country development	Continuous: following information reported in hdr.undp.org/en/content/human-development-index-hdi	Mean (±SE): 0.86 (±0.004)
Attribute	Authorization needed to fish within the MPA	Formal authorization to be required by the fishermen to MPA management board in order to perform fishing within the MPA	Binary: Yes(1)/No(0)	Frequency: 64%
Attribute	Enforcement	Efficacy and the effort provided by the reserve personnel, the coast guard	Categorical: 3 levels (from 1 to 3) following Guidetti et al. 2008, Sala et al. 2012, Edgar et al. 2014	Median: 2

		or other marine police forces in doing an active surveillance against illegal activities		
Attribute	MPA implementation year	Year of formal implementation of the MPA	Continuous	Mean (±SE): 1991 (±2)
Attribute	MPA no-take zone area	Coverage of the no-take area (i.e. where extractive activities, including fishing, are forbidden)	Continuous, in km^2	Mean (±SE): 10 (±5)
Attribute	MPA total area	Total coverage of the MPA	Continuous, in km^2	Mean (±SE): 8126 (±37)
Attribute	Number of artisanal vessels fishing within the MPA	Number of vessels fishing within the MPA, as a proxy of fishing effort	Continuous	Mean (±SE): 42 (±8)
Attribute	*Numerus clausus*	Presence of a maximum number of authorizations to fish within the MPA set *a priori* by the MPA management body	Binary: Yes(1)/No(0)	Frequency: 12%
Attribute	Only local fishermen allowed to fish within the MPA	Only fishermen resident within the MPA area or neighboring areas are allowed to perform SSF within the MPA	Binary: Yes(1)/No(0)	Frequency: 60%
Attribute	Leader among fishermen	Presence of an influential person among fishermen, recognized as a leader that can act as a liaison with the MPA management body	Binary: Yes(1)/No(0)	Frequency: 44%
Attribute	Presence of a management plan	Formal or informal arrangement between MPA management body and fishermen which details the agreed objectives for the fishery and specifies the management rules and regulations which apply to it	Binary: Yes(1)/No(0)	Frequency: 68%
Attribute	Presence of an incentive promoting sustainable	The MPA puts in action specific programs and activities (e.g. seafood awareness campaigns,	Binary: Yes(1)/No(0)	Frequency: 44%

	fishing	MPA-labeling) to promoting sustainable fishing within		
Attribute	Presence of fishermen in the MPA board	Formal inclusion of fishermen representative(s) having decisional power within the MPA management board	Binary: Yes(1)/No(0)	Frequency: 52%
Attribute	Ratio no-take area/total area	Ratio coverage of MPA no-take area/ total MPA coverage	Continuous	Mean (±SE): 0.11 (±0.03)
Attribute	Ratio artisanal vessels fishing within the MPA/MPA total area	Ratio of the number of artisanal vessels fishing within the MPA/ total MPA coverage	Continuous	Mean (±SE): 1.8 (±0.65)
Attribute	Recreational fishing allowed within the MPA	Recreational fishermen are allowed to fish within the MPA	Binary: Yes(1)/No(0)	Frequency: 72%
Attribute	Fishermen engagement level	The level of engagement of artisanal fishermen into management of artisanal fishing in MPA	Categorical: 5 levels (from 0 to 4) following Di Franco et al. 2014 (see details in "database compilation")	Median: 3
Outcome	Ecological effectiveness	Fish density/biomass increases as a result of the implementation of MPA (before-after analysis) or when compared with open access areas (control-impact analysis).	Binary: Yes(1)/No(0)	Frequency: 64%
Outcome	Fishermen incomes	Fishermen incomes increase as a result of the implementation of the MPA (before-after analysis) or when compared with open access areas (control-impact analysis).	Binary: Yes(1)/No(0)	Frequency: 68%
Outcome	Add-on stewardship benefits	The direct and indirect potential benefits resulting from fishermen environmental stewardship (i.e. commitment into MPA SSF sustainable management practices and	Binary: Yes(1)/No(0)	Frequency: 60%

	participation to research and environmental programs)	

Table S3. Minimum number of MPAs from which the attribute was confirmed in at least 950 out of 1000 iterations.

Attributes	Number of MPAs
Fishermen engagement level	20
Presence of fishermen in the board	21
Enforcement level	21
Presence of activity promoting sustainable fishing	22
Management plan	22
HDI	22

Table S4. Number of MPAs whose success score may be changed a) ±1, b) ± 1 or 2 with no significant effect on attribute's decision (i.e. attribute still confirmed in at least 950 out of 1000 iterations). Attributes are listed following decreasing number of MPAs.

a)

**Attributes**	**Number of MPAs**
Enforcement level	9
Fishermen engagement level	6
Management plan	5
Presence of fishermen in the board	1
Presence of activity promoting sustainable fishing	1
HDI	0

b)

**Attributes**	**Number of MPAs**
Fishermen engagement level	5
Enforcement level	4
Management plan	3
Presence of fishermen in the board	2
Presence of activity promoting sustainable fishing	2
HDI	0

Supplementary figures

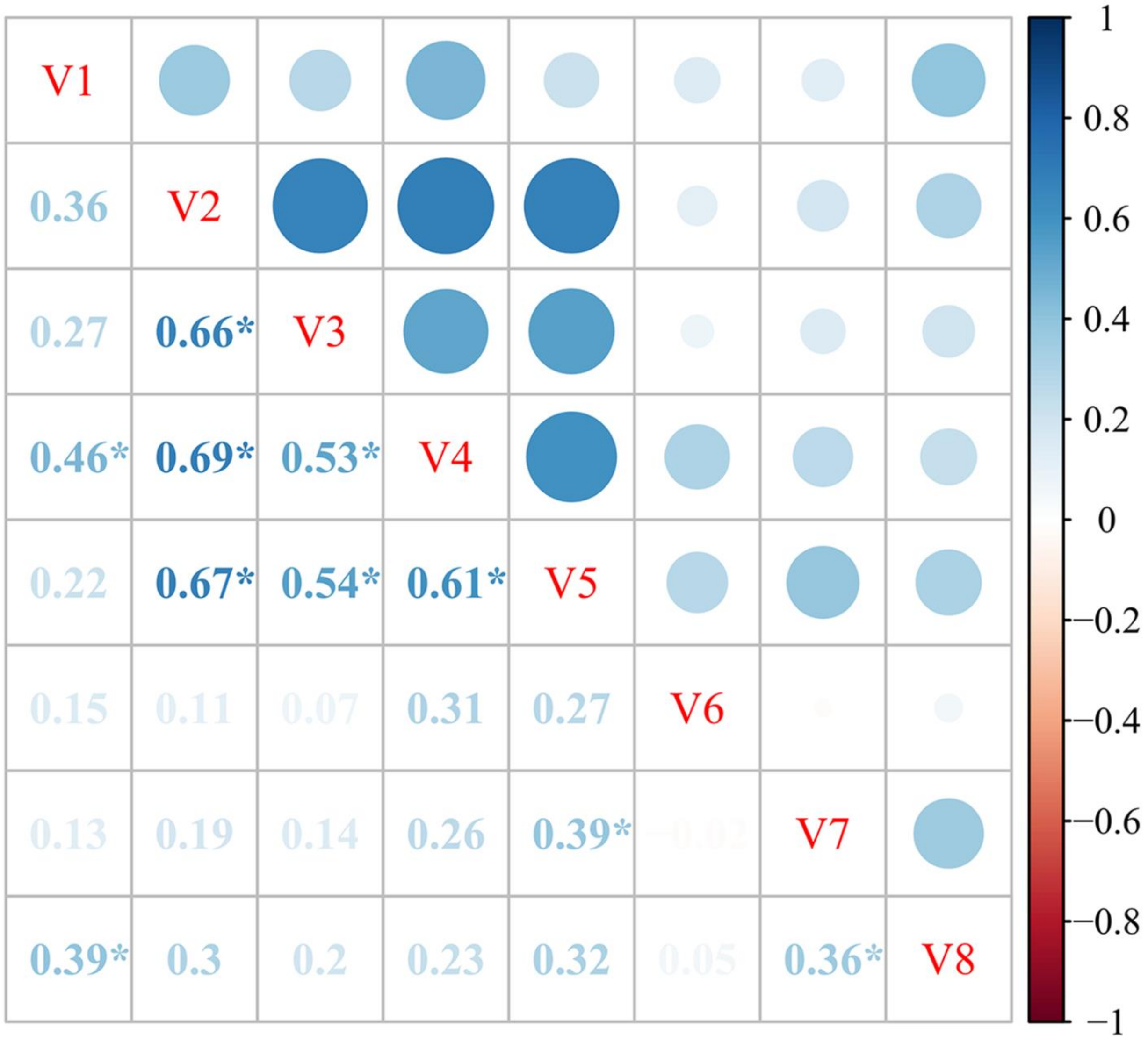

Figure S1. Correlation matrices for the 8 most important attributes (V) across the 25 case studies. Bold values indicate correlation coefficients r > 0.60. Asterisks indicate significant correlation among the two variables. Bubble size is proportional to r value and colors denote magnitude of the correlation coefficients from red (negative) to blue (positive). Variable numeration (Vn) follow the order of the 8 most important attributes in Fig. 2: V1= MPA enforcement, V2= fishermen engagement level, V3= presence of fishermen within the management board, V4=

presence of an incentive promoting sustainable fishing, V5= presence of a management plan for SSF, V6= HDI (human development index, a proxy for country development), V7= the portion of each MPA covered by no-take zone, V8= the restriction of fishing rights exclusively to local fishermen.

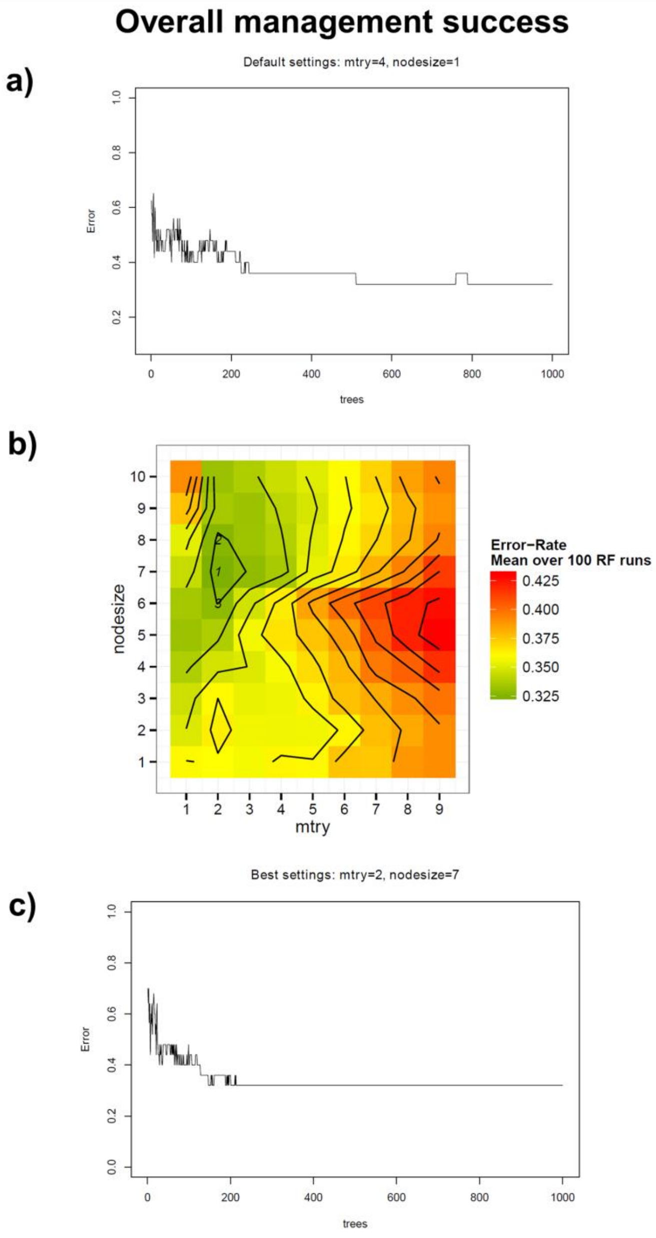

Figure S2. Optimization of the RF model for the outcome Overall Management Success. a) plot of the effect of ntree on out-of bag error-rate by using default settings (mtry and nodesize); b) grid search showing the error rate (mean over 100 RF runs) for each combination of mtry and nodesize; c) plot of the effect of ntree on out-of bag error-rate by using best combination of mtry and nodesize found using the grid search

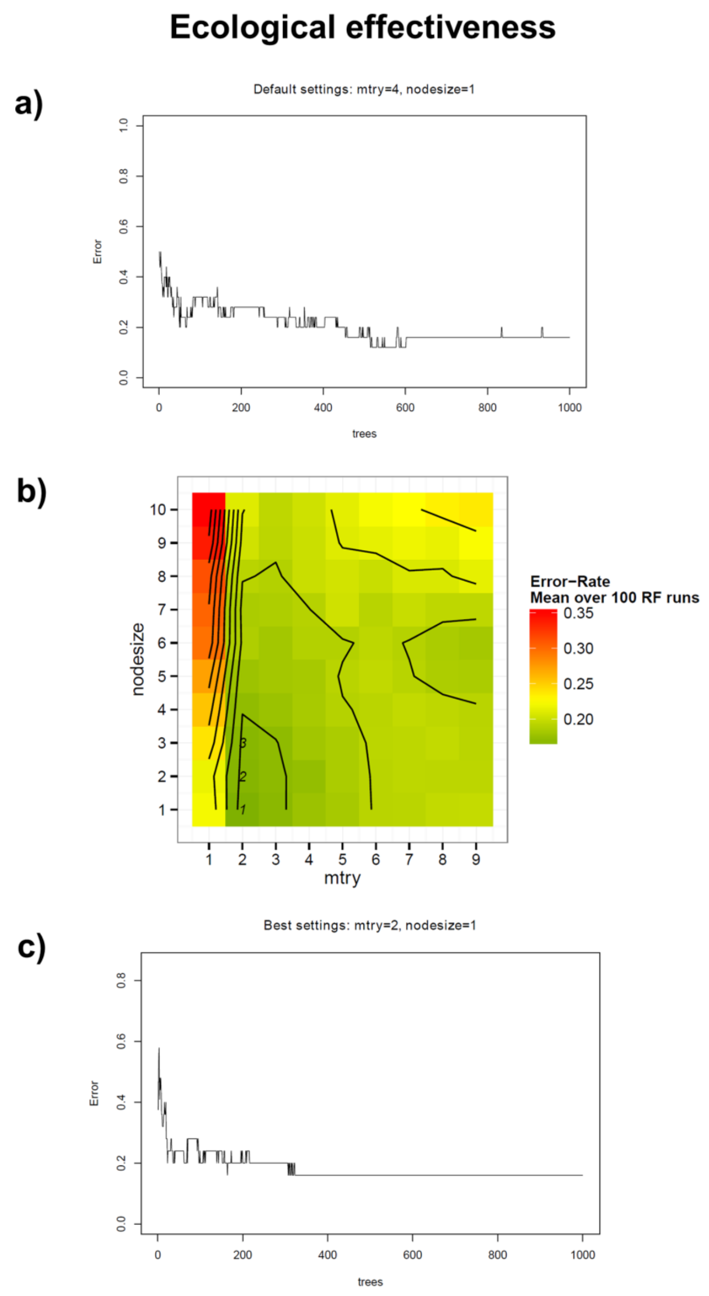

Figure S3. Optimization of the RF model for the outcome Ecological Effectiveness. a) plot of the effect of ntree on out-of bag error-rate by using default settings (mtry and nodesize); b) grid search showing the error rate (mean over 100 RF runs) for each combination of mtry and nodesize; c) plot of the effect of ntree on out-of bag error-rate by using best combination of mtry and nodesize found using the grid search

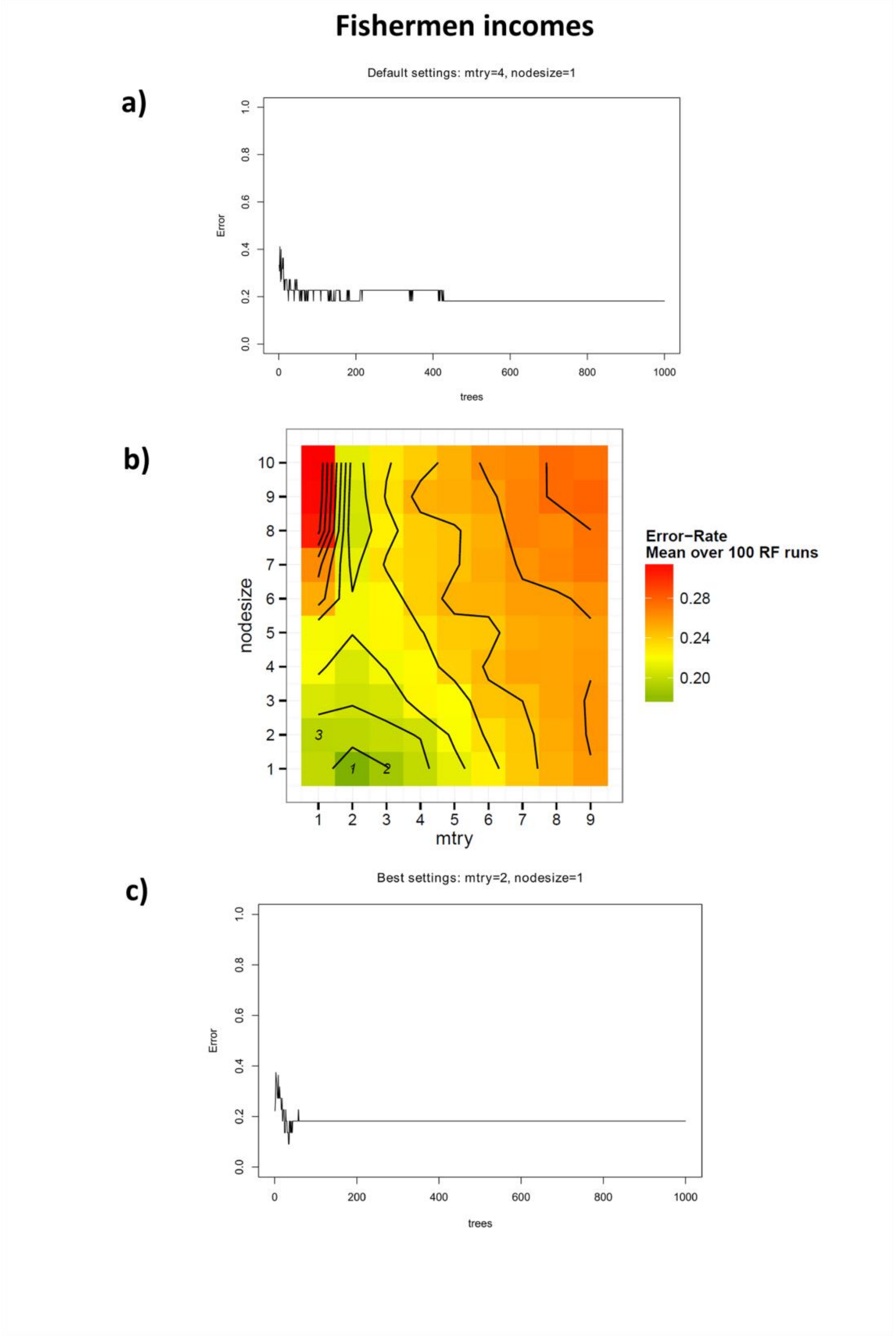

Figure S4. Optimization of the RF model for the outcome fishermen incomes. a) plot of the effect of ntree on out-of bag error-rate by using default settings (mtry and nodesize); b) grid search showing the error rate (mean over 100 RF runs) for each combination of mtry and nodesize; c) plot of the effect of ntree on out-of bag error-rate by using best combination of mtry and nodesize found using the grid search

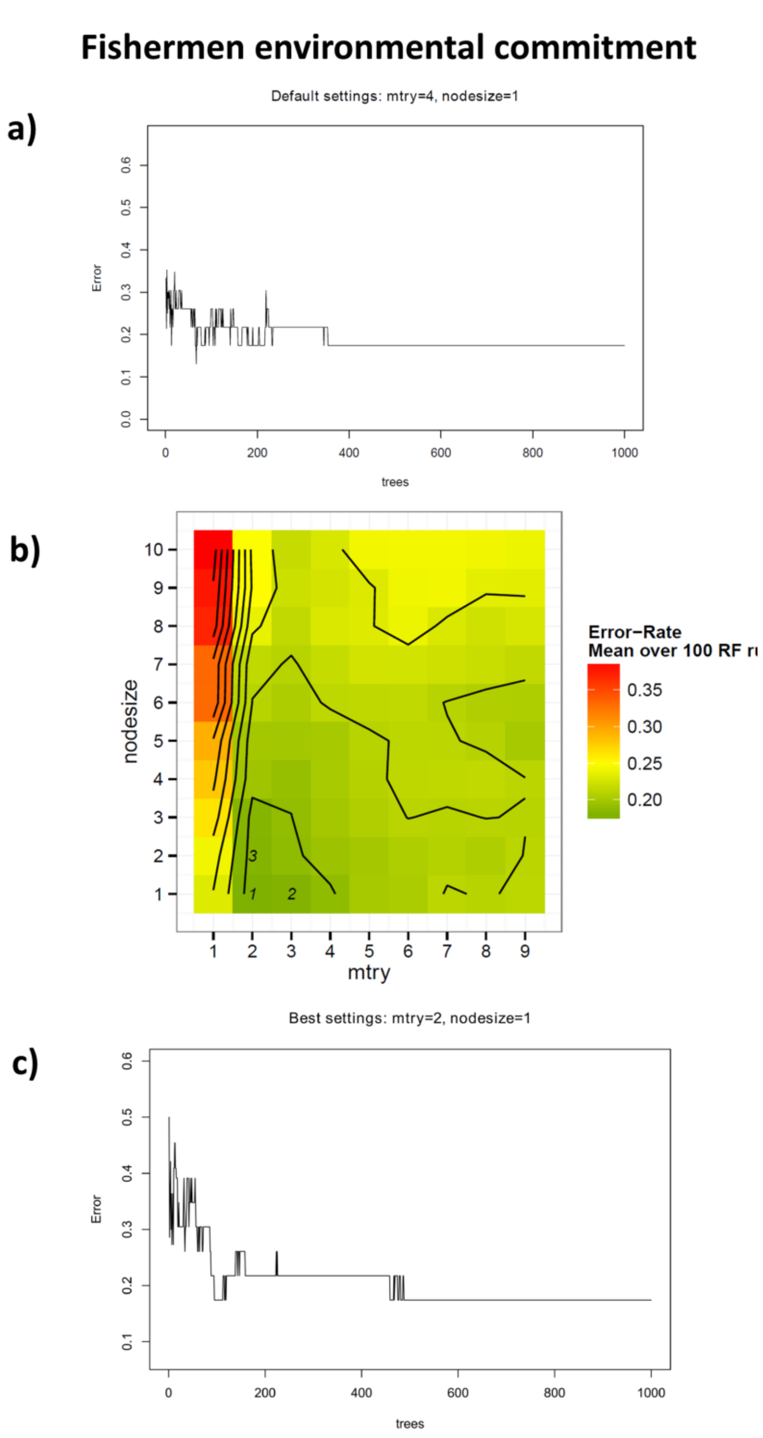

Figure S5. Optimization of the RF model for the outcome fishermen environmental commitment. a) plot of the effect of ntree on out-of bag error-rate by using default settings (mtry and nodesize); b) grid search showing the error rate (mean over 100 RF runs) for each combination of mtry and nodesize; c) plot of the effect of ntree on out-of bag error-rate by using best combination of mtry and nodesize found using the grid search

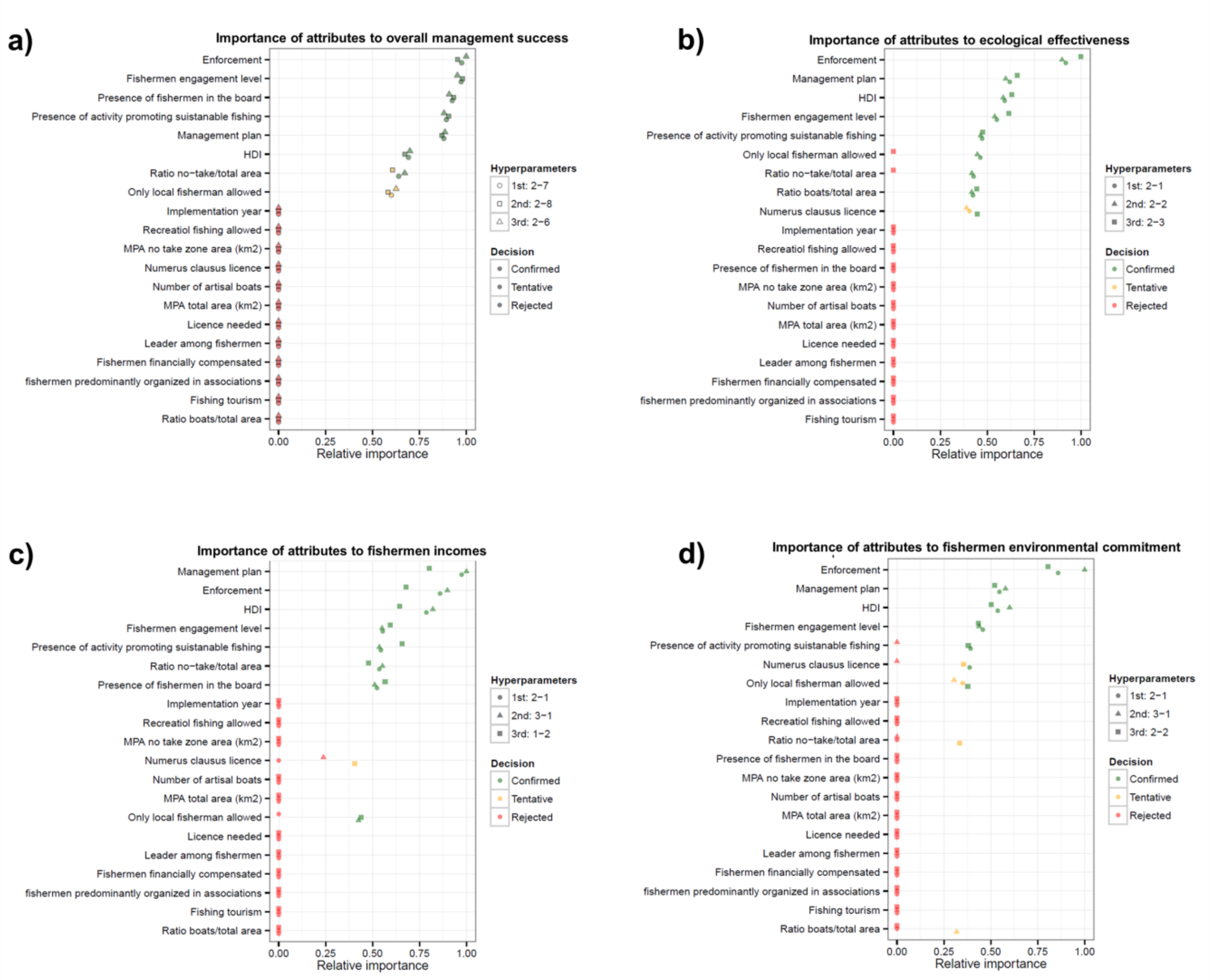

Figure S6. Graphical comparison of Boruta outputs based on different hyperparameters selection for each of the considered outcome: a) overall management success, b) ecological effectiveness, c) fishermen incomes, d) fishermen environmental commitment.

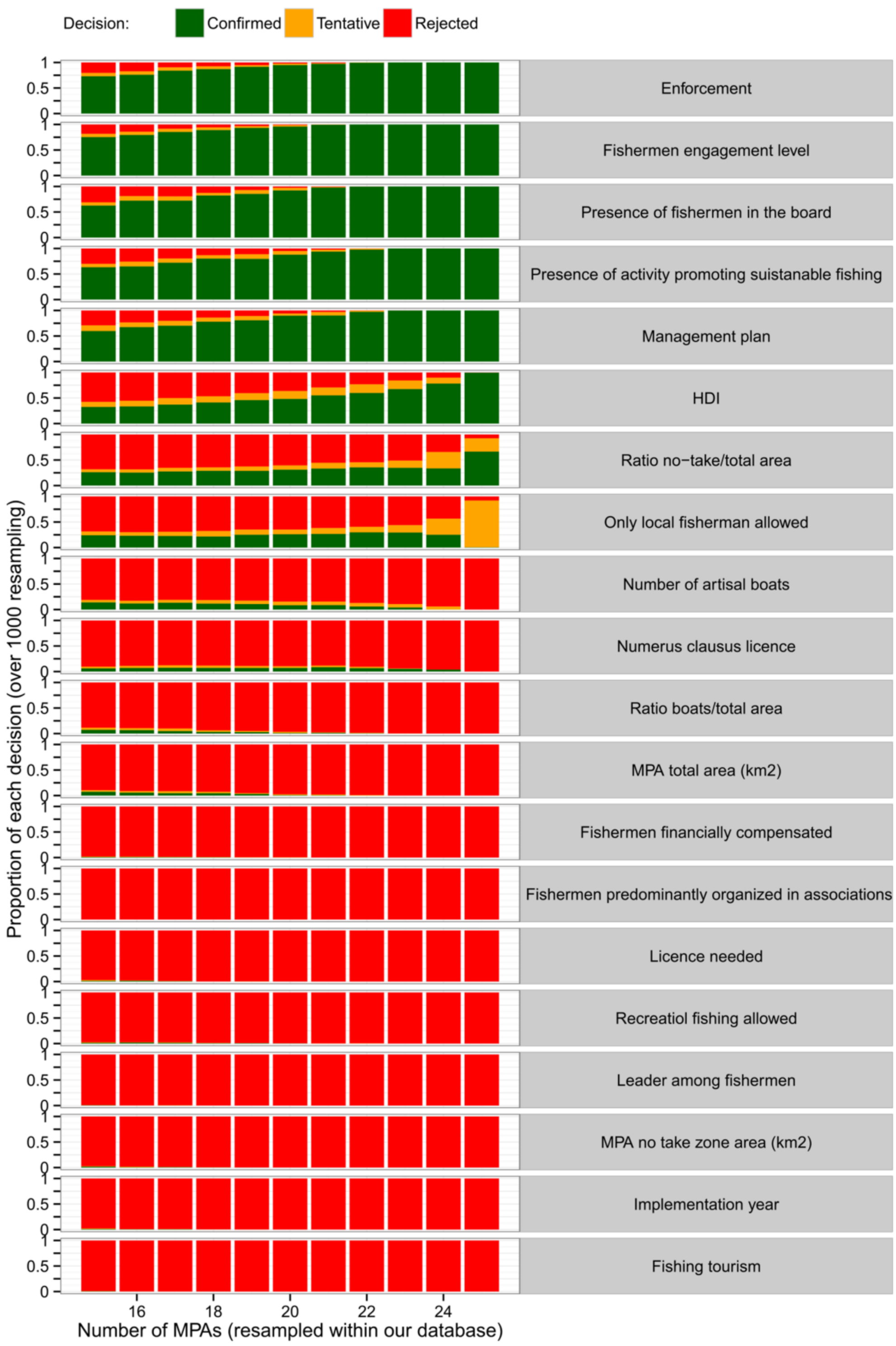

Figure S7. Proportion of Boruta decision (i.e. confirmed, tentative or rejected) for each of the 20 considered attributes at increasing number of MPAs where success score was modified by randomly summing or subtracting 1. Attributes are listed following the order of relevance highlighted in the main study (see Fig. 2).

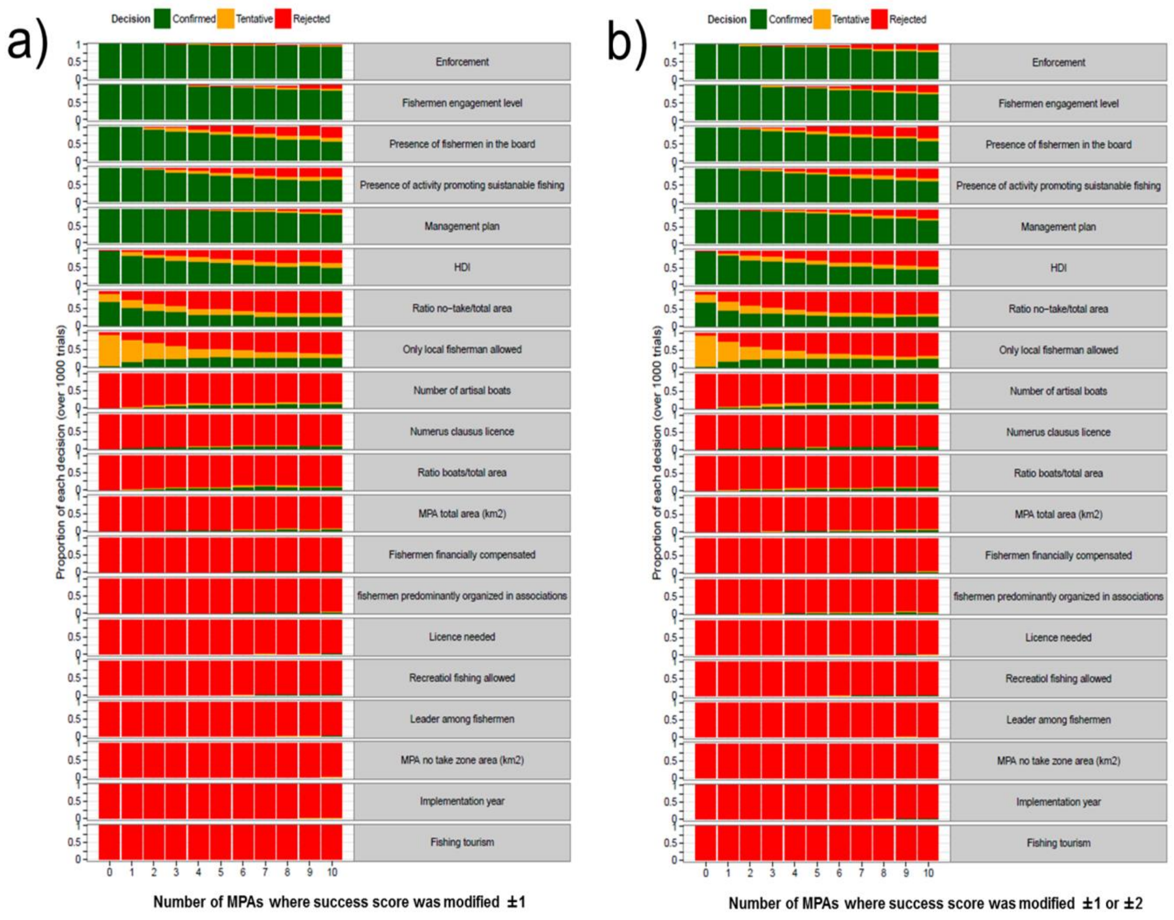

Figure S8. Proportion of Boruta decision (i.e. confirmed, tentative or rejected) for each of the 20 considered attributes at increasing number of MPAs where success score was modified by a) randomly summing or subtracting 1, b) randomly summing or subtracting 1 or 2. Attributes are listed following the order of relevance highlighted in the main study (see Fig. 2).
